# Supplementary material for: A novel long noncoding RNA (lncRNA), LINC02657(LASTR), is a prognostic biomarker associated with immune infiltrates of lung adenocarcinoma based on unsupervised cluster analysis
Source: PeerJ. 2023 Nov 28;11:e16167. doi: 10.7717/peerj.16167 (PMC10691363; doi:10.7717/peerj.16167)
Supplement: File S1 [file peerj-11-16167-s003.docx]

**Supplementary Materials. LASTR expression and overall survival information of lung adenocarcinoma patients included in the survival prognosis study in the TCGA-LUAD database.**

| **sample_id** | **OS_event** | **OS_time** | **LASTR** |
| --- | --- | --- | --- |
| TCGA-05-4244-01A-01R-1107-07 | Alive | 0 | 2.0654 |
| TCGA-05-4249-01A-01R-1107-07 | Alive | 1523 | 0.3042 |
| TCGA-05-4250-01A-01R-1107-07 | Dead | 121 | 1.8618 |
| TCGA-05-4382-01A-01R-1206-07 | Alive | 607 | 0.9497 |
| TCGA-05-4384-01A-01R-1755-07 | Alive | 426 | 2.3423 |
| TCGA-05-4389-01A-01R-1206-07 | Alive | 1369 | 0.4150 |
| TCGA-05-4390-01A-02R-1755-07 | Alive | 1126 | 0.9261 |
| TCGA-05-4395-01A-01R-1206-07 | Dead | 0 | 1.4636 |
| TCGA-05-4396-01A-21R-1858-07 | Dead | 303 | 1.2167 |
| TCGA-05-4397-01A-01R-1206-07 | Dead | 731 | 1.0813 |
| TCGA-05-4398-01A-01R-1206-07 | Alive | 1431 | 2.3982 |
| TCGA-05-4402-01A-01R-1206-07 | Dead | 244 | 1.1688 |
| TCGA-05-4403-01A-01R-1206-07 | Alive | 578 | 1.1254 |
| TCGA-05-4405-01A-21R-1858-07 | Alive | 610 | 1.3775 |
| TCGA-05-4410-01A-21R-1858-07 | Alive | 0 | 1.0786 |
| TCGA-05-4415-01A-22R-1858-07 | Dead | 91 | 0.2425 |
| TCGA-05-4417-01A-22R-1858-07 | Alive | 455 | 0.6226 |
| TCGA-05-4418-01A-01R-1206-07 | Dead | 274 | 0.4425 |
| TCGA-05-4420-01A-01R-1206-07 | Alive | 912 | 0.5470 |
| TCGA-05-4422-01A-01R-1206-07 | Alive | 365 | 0.2425 |
| TCGA-05-4424-01A-22R-1858-07 | Alive | 913 | 0.3233 |
| TCGA-05-4425-01A-01R-1755-07 | Alive | 669 | 0.7388 |
| TCGA-05-4426-01A-01R-1206-07 | Alive | 791 | 0.8474 |
| TCGA-05-4427-01A-21R-1858-07 | Alive | 791 | 2.6105 |
| TCGA-05-4430-01A-02R-1206-07 | Alive | 761 | 0.4238 |
| TCGA-05-4432-01A-01R-1206-07 | Alive | 761 | 0.2297 |
| TCGA-05-4433-01A-22R-1858-07 | Alive | 730 | 0.8429 |
| TCGA-05-4434-01A-01R-1206-07 | Dead | 457 | 2.1320 |
| TCGA-05-5420-01A-01R-1628-07 | Alive | 457 | 0.1418 |
| TCGA-05-5423-01A-01R-1628-07 | Alive | 151 | 0.2410 |
| TCGA-05-5425-01A-02R-1628-07 | Alive | 882 | 0.1254 |
| TCGA-05-5428-01A-01R-1628-07 | Alive | 670 | 0.3362 |
| TCGA-05-5429-01A-01R-1628-07 | Dead | 275 | 0.4482 |
| TCGA-05-5715-01A-01R-1628-07 | Alive | 62 | 0.1516 |
| TCGA-35-3615-01A-01R-0946-07 | Alive | 14 | 0.1656 |
| TCGA-35-4122-01A-01R-1107-07 | Alive | 225 | 1.4638 |
| TCGA-35-4123-01A-01R-1107-07 | Alive | 182 | 1.6099 |
| TCGA-35-5375-01A-01R-1628-07 | Alive | 264 | 0.1206 |
| TCGA-38-4625-01A-01R-1206-07 | Alive | 2973 | 1.8398 |
| TCGA-38-4626-01A-01R-1206-07 | Alive | 3674 | 0.1274 |
| TCGA-38-4627-01A-01R-1206-07 | Dead | 1147 | 0.6014 |
| TCGA-38-4628-01A-01R-1206-07 | Dead | 1492 | 0.1337 |
| TCGA-38-4629-01A-02R-1206-07 | Dead | 864 | 2.3705 |
| TCGA-38-4630-01A-01R-1206-07 | Dead | 1073 | 0.1074 |
| TCGA-38-4631-01A-01R-1755-07 | Dead | 354 | 0.6002 |
| TCGA-38-4632-01A-01R-1755-07 | Dead | 1357 | 1.6057 |
| TCGA-38-6178-01A-11R-1755-07 | Alive | 448 | 0.4931 |
| TCGA-38-7271-01A-11R-2039-07 | Dead | 800 | 0.1189 |
| TCGA-38-A44F-01A-11R-A24H-07 | Alive | 133 | 1.2097 |
| TCGA-44-2655-01A-01R-0946-07 | Alive | 1324 | 1.2921 |
| TCGA-44-2656-01A-02R-0946-07 | Alive | 1429 | 0.4398 |
| TCGA-44-2656-01A-02R-A278-07 | Alive | 1429 | 0.3456 |
| TCGA-44-2656-01B-06R-A277-07 | Alive | 1429 | 0.8706 |
| TCGA-44-2657-01A-01R-1107-07 | Alive | 1351 | 0.0366 |
| TCGA-44-2659-01A-01R-0946-07 | Alive | 1367 | 0.3645 |
| TCGA-44-2661-01A-01R-1107-07 | Alive | 1159 | 0.0783 |
| TCGA-44-2662-01A-01R-0946-07 | Alive | 1280 | 0.5105 |
| TCGA-44-2662-01A-01R-A278-07 | Alive | 1280 | 0.1809 |
| TCGA-44-2662-01B-02R-A277-07 | Alive | 1280 | 0.7509 |
| TCGA-44-2665-01A-01R-0946-07 | Alive | 1301 | 2.0898 |
| TCGA-44-2665-01B-06R-A277-07 | Alive | 1301 | 2.0244 |
| TCGA-44-2666-01A-01R-0946-07 | Dead | 97 | 0.2766 |
| TCGA-44-2666-01A-01R-A278-07 | Dead | 97 | 0.2011 |
| TCGA-44-2666-01B-02R-A277-07 | Dead | 97 | 1.1397 |
| TCGA-44-2668-01A-01R-0946-07 | Dead | 761 | 1.4909 |
| TCGA-44-2668-01A-01R-A278-07 | Dead | 761 | 0.7865 |
| TCGA-44-2668-01B-02R-A277-07 | Dead | 761 | 2.6229 |
| TCGA-44-3396-01A-01R-1206-07 | Alive | 1130 | 1.3456 |
| TCGA-44-3398-01A-01R-1107-07 | Alive | 1163 | 0.7353 |
| TCGA-44-3917-01A-01R-A278-07 | Alive | 1183 | 0.8570 |
| TCGA-44-3917-01B-02R-A277-07 | Alive | 1183 | 2.4476 |
| TCGA-44-3918-01A-01R-1107-07 | Alive | 1036 | 1.5287 |
| TCGA-44-3918-01A-01R-A278-07 | Alive | 1036 | 1.5759 |
| TCGA-44-3918-01B-02R-A277-07 | Alive | 1036 | 3.4275 |
| TCGA-44-3919-01A-02R-1107-07 | Dead | 1026 | 0.7176 |
| TCGA-44-4112-01A-01R-1107-07 | Dead | 808 | 1.7191 |
| TCGA-44-4112-01B-06R-A277-07 | Dead | 808 | 2.2103 |
| TCGA-44-5643-01A-01R-1628-07 | Alive | 1013 | 0.5528 |
| TCGA-44-5644-01A-21R-2039-07 | Alive | 863 | 1.1701 |
| TCGA-44-5645-01A-01R-1628-07 | Alive | 852 | 0.0283 |
| TCGA-44-5645-01A-01R-A278-07 | Alive | 852 | 0.0672 |
| TCGA-44-5645-01B-04R-A277-07 | Alive | 852 | 0.2147 |
| TCGA-44-6145-01A-11R-1755-07 | Alive | 595 | 0.8025 |
| TCGA-44-6146-01A-11R-1755-07 | Alive | 728 | 0.9528 |
| TCGA-44-6146-01A-11R-A278-07 | Alive | 728 | 0.4009 |
| TCGA-44-6146-01B-04R-A277-07 | Alive | 728 | 1.5899 |
| TCGA-44-6147-01A-11R-1755-07 | Alive | 845 | 0.3529 |
| TCGA-44-6147-01A-11R-A278-07 | Alive | 845 | 0.1601 |
| TCGA-44-6147-01B-06R-A277-07 | Alive | 845 | 0.4518 |
| TCGA-44-6148-01A-11R-1755-07 | Alive | 704 | 0.3063 |
| TCGA-44-6774-01A-21R-1858-07 | Alive | 658 | 0.2780 |
| TCGA-44-6775-01A-11R-1858-07 | Alive | 705 | 0.4052 |
| TCGA-44-6775-01A-11R-A278-07 | Alive | 705 | 0.3858 |
| TCGA-44-6775-01C-02R-A277-07 | Alive | 705 | 0.5124 |
| TCGA-44-6776-01A-11R-1858-07 | Alive | 2616 | 0.0000 |
| TCGA-44-6777-01A-11R-1858-07 | Dead | 987 | 0.5721 |
| TCGA-44-6778-01A-11R-1858-07 | Alive | 1864 | 0.7378 |
| TCGA-44-6779-01A-11R-1858-07 | Dead | 500 | 0.6504 |
| TCGA-44-7659-01A-11R-2066-07 | Alive | 691 | 0.2279 |
| TCGA-44-7660-01A-11R-2066-07 | Alive | 592 | 0.4817 |
| TCGA-44-7661-01A-11R-2066-07 | Dead | 557 | 3.2624 |
| TCGA-44-7662-01A-11R-2066-07 | Alive | 218 | 2.3676 |
| TCGA-44-7667-01A-31R-2066-07 | Alive | 1097 | 0.5776 |
| TCGA-44-7669-01A-21R-2066-07 | Dead | 574 | 0.2504 |
| TCGA-44-7670-01A-11R-2066-07 | Alive | 882 | 0.1058 |
| TCGA-44-7671-01A-11R-2066-07 | Alive | 889 | 0.7624 |
| TCGA-44-7672-01A-11R-2066-07 | Alive | 719 | 0.9337 |
| TCGA-44-8117-01A-11R-2241-07 | Alive | 385 | 1.9216 |
| TCGA-44-8119-01A-11R-2241-07 | Alive | 285 | 0.5550 |
| TCGA-44-8120-01A-11R-2241-07 | Alive | 260 | 0.9570 |
| TCGA-44-A479-01A-31R-A24H-07 | Alive | 486 | 0.2223 |
| TCGA-44-A47A-01A-21R-A24H-07 | Alive | 466 | 0.9467 |
| TCGA-44-A47B-01A-11R-A24H-07 | Alive | 287 | 0.1400 |
| TCGA-44-A47G-01A-21R-A24H-07 | Alive | 351 | 0.4873 |
| TCGA-44-A4SS-01A-11R-A24X-07 | Alive | 415 | 0.9865 |
| TCGA-44-A4SU-01A-11R-A24X-07 | Dead | 409 | 1.1781 |
| TCGA-49-4486-01A-01R-1206-07 | Dead | 2318 | 0.2639 |
| TCGA-49-4487-01A-21R-1858-07 | Dead | 855 | 0.2457 |
| TCGA-49-4488-01A-01R-1755-07 | Dead | 869 | 0.7906 |
| TCGA-49-4490-01A-21R-1858-07 | Dead | 385 | 1.1019 |
| TCGA-49-4494-01A-01R-1206-07 | Dead | 1081 | 1.4080 |
| TCGA-49-4501-01A-01R-1206-07 | Dead | 1421 | 0.2460 |
| TCGA-49-4505-01A-01R-1206-07 | Dead | 428 | 1.1956 |
| TCGA-49-4506-01A-01R-1206-07 | Dead | 999 | 0.2457 |
| TCGA-49-4507-01A-01R-1206-07 | Dead | 268 | 0.6990 |
| TCGA-49-4510-01A-01R-1206-07 | Dead | 896 | 0.5474 |
| TCGA-49-4512-01A-21R-1858-07 | Dead | 905 | 0.2534 |
| TCGA-49-4514-01A-21R-1858-07 | Alive | 1700 | 0.2318 |
| TCGA-49-6742-01A-11R-1858-07 | Dead | 488 | 0.5636 |
| TCGA-49-6743-01A-11R-1858-07 | Alive | 1621 | 0.3288 |
| TCGA-49-6744-01A-11R-1858-07 | Alive | 1683 | 0.2579 |
| TCGA-49-6745-01A-11R-1858-07 | Alive | 522 | 1.9395 |
| TCGA-49-6761-01A-31R-1949-07 | Alive | 354 | 3.0802 |
| TCGA-49-6767-01A-11R-1858-07 | Alive | 677 | 1.9609 |
| TCGA-49-AAQV-01A-11R-A39D-07 | Dead | 677 | 1.0762 |
| TCGA-49-AAR0-01A-21R-A39D-07 | Alive | 4765 | 0.3984 |
| TCGA-49-AAR2-01A-11R-A39D-07 | Alive | 2224 | 1.1324 |
| TCGA-49-AAR3-01A-11R-A41B-07 | Alive | 1893 | 1.1473 |
| TCGA-49-AAR4-01A-12R-A41B-07 | Dead | 879 | 1.7190 |
| TCGA-49-AAR9-01A-21R-A41B-07 | Dead | 260 | 1.1759 |
| TCGA-49-AARE-01A-11R-A41B-07 | Dead | 1229 | 1.2719 |
| TCGA-49-AARN-01A-21R-A41B-07 | Dead | 1135 | 1.4759 |
| TCGA-49-AARO-01A-12R-A41B-07 | Alive | 3759 | 2.0622 |
| TCGA-49-AARQ-01A-11R-A41B-07 | Alive | 6732 | 0.0685 |
| TCGA-49-AARR-01A-11R-A41B-07 | Alive | 4992 | 0.5019 |
| TCGA-4B-A93V-01A-11R-A39D-07 | Dead | 300 | 2.3191 |
| TCGA-50-5044-01A-21R-1858-07 | Dead | 624 | 0.8287 |
| TCGA-50-5045-01A-01R-1628-07 | Dead | 2174 | 0.5518 |
| TCGA-50-5049-01A-01R-1628-07 | Alive | 3094 | 0.0636 |
| TCGA-50-5051-01A-21R-1858-07 | Dead | 478 | 1.2705 |
| TCGA-50-5055-01A-01R-1628-07 | Dead | 1830 | 0.1598 |
| TCGA-50-5066-01A-01R-1628-07 | Alive | 1442 | 0.9230 |
| TCGA-50-5066-02A-11R-2090-07 | Alive | 1442 | 0.2776 |
| TCGA-50-5068-01A-01R-1628-07 | Dead | 1499 | 0.3746 |
| TCGA-50-5072-01A-21R-1858-07 | Dead | 250 | 0.8286 |
| TCGA-50-5930-01A-11R-1755-07 | Dead | 282 | 0.2940 |
| TCGA-50-5931-01A-11R-1755-07 | Dead | 434 | 0.8249 |
| TCGA-50-5932-01A-11R-1755-07 | Dead | 1235 | 0.5578 |
| TCGA-50-5933-01A-11R-1755-07 | Dead | 2393 | 1.7129 |
| TCGA-50-5935-01A-11R-1755-07 | Dead | 653 | 0.8239 |
| TCGA-50-5936-01A-11R-1628-07 | Dead | 257 | 0.4529 |
| TCGA-50-5939-01A-11R-1628-07 | Dead | 460 | 0.4816 |
| TCGA-50-5941-01A-11R-1755-07 | Alive | 1474 | 0.7322 |
| TCGA-50-5942-01A-21R-1755-07 | Alive | 1847 | 0.2664 |
| TCGA-50-5944-01A-11R-1755-07 | Alive | 1750 | 0.2196 |
| TCGA-50-5946-01A-11R-1755-07 | Alive | 1617 | 2.1815 |
| TCGA-50-5946-02A-11R-2090-07 | Alive | 1617 | 1.1986 |
| TCGA-50-6590-01A-12R-1858-07 | Dead | 1288 | 0.6081 |
| TCGA-50-6591-01A-11R-1755-07 | Dead | 119 | 0.3875 |
| TCGA-50-6592-01A-11R-1755-07 | Dead | 777 | 1.1906 |
| TCGA-50-6593-01A-11R-1755-07 | Dead | 336 | 0.7696 |
| TCGA-50-6594-01A-11R-1755-07 | Dead | 370 | 1.4409 |
| TCGA-50-6595-01A-12R-1858-07 | Dead | 189 | 0.9737 |
| TCGA-50-6597-01A-11R-1858-07 | Dead | 1268 | 0.0756 |
| TCGA-50-6673-01A-11R-1949-07 | Dead | 22 | 0.6195 |
| TCGA-50-7109-01A-11R-2039-07 | Dead | 308 | 0.2113 |
| TCGA-50-8457-01A-11R-2326-07 | Alive | 1125 | 0.1677 |
| TCGA-50-8459-01A-11R-2326-07 | Alive | 1119 | 1.1601 |
| TCGA-50-8460-01A-11R-2326-07 | Alive | 829 | 0.2056 |
| TCGA-53-7624-01A-11R-2066-07 | Dead | 1043 | 0.0879 |
| TCGA-53-7626-01A-12R-2066-07 | Dead | 929 | 0.2713 |
| TCGA-53-7813-01A-11R-2170-07 | Alive | 424 | 0.3889 |
| TCGA-53-A4EZ-01A-12R-A24X-07 | Alive | 1071 | 0.6863 |
| TCGA-55-1592-01A-01R-0946-07 | Dead | 701 | 0.5800 |
| TCGA-55-1594-01A-01R-0946-07 | Alive | 1178 | 0.3602 |
| TCGA-55-1595-01A-01R-0946-07 | Alive | 1479 | 1.1419 |
| TCGA-55-1596-01A-01R-0946-07 | Alive | 2065 | 0.9010 |
| TCGA-55-5899-01A-11R-1628-07 | Alive | 930 | 1.5923 |
| TCGA-55-6543-01A-11R-1755-07 | Alive | 435 | 0.5504 |
| TCGA-55-6642-01A-11R-1858-07 | Alive | 2449 | 0.5919 |
| TCGA-55-6712-01A-11R-1858-07 | Dead | 171 | 0.7389 |
| TCGA-55-6968-01A-11R-1949-07 | Dead | 1293 | 0.3998 |
| TCGA-55-6969-01A-11R-1949-07 | Alive | 1239 | 0.8843 |
| TCGA-55-6970-01A-11R-1949-07 | Dead | 464 | 0.4160 |
| TCGA-55-6971-01A-11R-1949-07 | Alive | 1400 | 1.5463 |
| TCGA-55-6972-01A-11R-1949-07 | Dead | 1632 | 0.2324 |
| TCGA-55-6975-01A-11R-1949-07 | Dead | 118 | 0.3286 |
| TCGA-55-6978-01A-11R-1949-07 | Dead | 176 | 2.0502 |
| TCGA-55-6979-01A-11R-1949-07 | Dead | 237 | 0.6946 |
| TCGA-55-6980-01A-11R-1949-07 | Alive | 2109 | 1.3359 |
| TCGA-55-6981-01A-11R-1949-07 | Dead | 1379 | 0.7619 |
| TCGA-55-6982-01A-11R-1949-07 | Dead | 995 | 0.3437 |
| TCGA-55-6983-01A-11R-1949-07 | Alive | 2823 | 1.1840 |
| TCGA-55-6984-01A-11R-1949-07 | Dead | 760 | 0.7938 |
| TCGA-55-6985-01A-11R-1949-07 | Alive | 1233 | 0.7721 |
| TCGA-55-6986-01A-11R-1949-07 | Alive | 3261 | 0.6931 |
| TCGA-55-6987-01A-11R-1949-07 | Alive | 2137 | 0.2292 |
| TCGA-55-7227-01A-11R-2039-07 | Dead | 952 | 0.7954 |
| TCGA-55-7281-01A-11R-2039-07 | Alive | 872 | 0.4529 |
| TCGA-55-7283-01A-11R-2039-07 | Alive | 609 | 0.3546 |
| TCGA-55-7284-01B-11R-2241-07 | Dead | 243 | 1.5945 |
| TCGA-55-7570-01A-11R-2039-07 | Alive | 824 | 0.4307 |
| TCGA-55-7573-01A-11R-2039-07 | Alive | 487 | 0.1768 |
| TCGA-55-7574-01A-11R-2039-07 | Dead | 995 | 0.9116 |
| TCGA-55-7576-01A-11R-2066-07 | Alive | 670 | 1.3458 |
| TCGA-55-7724-01A-11R-2170-07 | Alive | 705 | 0.6661 |
| TCGA-55-7725-01A-11R-2170-07 | Alive | 442 | 0.2396 |
| TCGA-55-7726-01A-11R-2170-07 | Alive | 652 | 2.7597 |
| TCGA-55-7727-01A-11R-2170-07 | Alive | 119 | 1.5710 |
| TCGA-55-7728-01A-11R-2187-07 | Alive | 704 | 1.4648 |
| TCGA-55-7815-01A-11R-2170-07 | Alive | 773 | 0.8217 |
| TCGA-55-7816-01A-11R-2170-07 | Dead | 468 | 0.2495 |
| TCGA-55-7903-01A-11R-2170-07 | Alive | 567 | 2.2224 |
| TCGA-55-7907-01A-11R-2170-07 | Dead | 343 | 0.7442 |
| TCGA-55-7910-01A-11R-2170-07 | Alive | 1040 | 1.1909 |
| TCGA-55-7911-01A-11R-2170-07 | Alive | 537 | 2.2084 |
| TCGA-55-7913-01B-11R-2241-07 | Dead | 561 | 1.6602 |
| TCGA-55-7914-01A-11R-2170-07 | Dead | 187 | 0.6251 |
| TCGA-55-7994-01A-11R-2187-07 | Alive | 603 | 2.5299 |
| TCGA-55-7995-01A-11R-2187-07 | Alive | 889 | 0.2696 |
| TCGA-55-8085-01A-11R-2241-07 | Alive | 904 | 0.5451 |
| TCGA-55-8087-01A-11R-2241-07 | Alive | 462 | 0.0436 |
| TCGA-55-8089-01A-11R-2241-07 | Dead | 702 | 2.3378 |
| TCGA-55-8090-01A-11R-2241-07 | Dead | 598 | 3.6378 |
| TCGA-55-8091-01A-11R-2241-07 | Alive | 600 | 1.0677 |
| TCGA-55-8092-01A-11R-2241-07 | Dead | 154 | 0.5753 |
| TCGA-55-8094-01A-11R-2241-07 | Alive | 541 | 1.5500 |
| TCGA-55-8096-01A-11R-2241-07 | Dead | 719 | 1.1500 |
| TCGA-55-8097-01A-11R-2241-07 | Alive | 476 | 0.2099 |
| TCGA-55-8203-01A-11R-2241-07 | Alive | 547 | 0.9899 |
| TCGA-55-8204-01A-11R-2241-07 | Alive | 515 | 1.7799 |
| TCGA-55-8205-01A-11R-2241-07 | Alive | 599 | 2.1320 |
| TCGA-55-8206-01A-11R-2241-07 | Alive | 888 | 0.1849 |
| TCGA-55-8207-01A-11R-2241-07 | Alive | 977 | 0.5167 |
| TCGA-55-8208-01A-11R-2241-07 | Alive | 674 | 0.1932 |
| TCGA-55-8299-01A-11R-2287-07 | Dead | 469 | 0.0696 |
| TCGA-55-8301-01A-11R-2287-07 | Alive | 534 | 0.5454 |
| TCGA-55-8302-01A-11R-2326-07 | Alive | 478 | 3.8070 |
| TCGA-55-8505-01A-11R-2403-07 | Alive | 440 | 0.7498 |
| TCGA-55-8506-01A-11R-2403-07 | Alive | 11 | 0.4852 |
| TCGA-55-8507-01A-11R-2403-07 | Alive | 418 | 0.6952 |
| TCGA-55-8508-01A-11R-2403-07 | Alive | 617 | 1.2313 |
| TCGA-55-8510-01A-11R-2403-07 | Alive | 539 | 0.3874 |
| TCGA-55-8511-01A-11R-2403-07 | Alive | 552 | 0.4906 |
| TCGA-55-8512-01A-11R-2403-07 | Dead | 607 | 0.4071 |
| TCGA-55-8513-01A-11R-2403-07 | Alive | 791 | 0.2989 |
| TCGA-55-8514-01A-11R-2403-07 | Alive | 520 | 0.1250 |
| TCGA-55-8614-01A-11R-2403-07 | Alive | 536 | 0.3160 |
| TCGA-55-8615-01A-11R-2403-07 | Alive | 446 | 3.0692 |
| TCGA-55-8616-01A-11R-2403-07 | Alive | 48 | 0.6104 |
| TCGA-55-8619-01A-11R-2403-07 | Alive | 416 | 0.3751 |
| TCGA-55-8620-01A-11R-2403-07 | Dead | 375 | 1.6441 |
| TCGA-55-8621-01A-11R-2403-07 | Alive | 515 | 0.6969 |
| TCGA-55-A48X-01A-11R-A24H-07 | Alive | 689 | 0.3341 |
| TCGA-55-A48Y-01A-11R-A24H-07 | Alive | 630 | 0.1924 |
| TCGA-55-A48Z-01A-12R-A24X-07 | Alive | 651 | 1.1594 |
| TCGA-55-A490-01A-11R-A466-07 | Dead | 99 | 1.1844 |
| TCGA-55-A491-01A-11R-A24H-07 | Alive | 626 | 1.0793 |
| TCGA-55-A492-01A-11R-A24H-07 | Alive | 596 | 0.7320 |
| TCGA-55-A493-01A-11R-A24H-07 | Alive | 28 | 1.3787 |
| TCGA-55-A494-01A-11R-A24X-07 | Alive | 481 | 2.2283 |
| TCGA-55-A4DF-01A-11R-A24H-07 | Dead | 614 | 0.6852 |
| TCGA-55-A4DG-01A-11R-A24H-07 | Alive | 608 | 0.6710 |
| TCGA-55-A57B-01A-12R-A39D-07 | Alive | 546 | 1.3105 |
| TCGA-62-8394-01A-11R-2326-07 | Dead | 139 | 1.3590 |
| TCGA-62-8395-01A-11R-2326-07 | Alive | 1216 | 0.5734 |
| TCGA-62-8397-01A-11R-2326-07 | Alive | 1289 | 0.1634 |
| TCGA-62-8398-01A-11R-2326-07 | Dead | 444 | 0.9589 |
| TCGA-62-8399-01A-21R-2326-07 | Alive | 2696 | 0.5440 |
| TCGA-62-8402-01A-11R-2326-07 | Dead | 1498 | 0.3250 |
| TCGA-62-A46O-01A-11R-A24H-07 | Dead | 1454 | 0.4264 |
| TCGA-62-A46P-01A-11R-A24H-07 | Dead | 594 | 0.0503 |
| TCGA-62-A46R-01A-11R-A24H-07 | Dead | 1725 | 1.1922 |
| TCGA-62-A46S-01A-11R-A24H-07 | Dead | 1653 | 0.5734 |
| TCGA-62-A46U-01A-11R-A24H-07 | Alive | 2067 | 2.0732 |
| TCGA-62-A46V-01A-11R-A24H-07 | Alive | 2199 | 1.4882 |
| TCGA-62-A46Y-01A-11R-A24H-07 | Dead | 414 | 0.7806 |
| TCGA-62-A470-01A-11R-A24H-07 | Dead | 1194 | 0.4722 |
| TCGA-62-A471-01A-12R-A24H-07 | Alive | 1246 | 1.0088 |
| TCGA-62-A472-01A-11R-A24H-07 | Alive | 910 | 3.8506 |
| TCGA-64-1676-01A-01R-0946-07 | Alive | 1728 | 0.4000 |
| TCGA-64-1677-01A-01R-0946-07 | Dead | 628 | 0.4976 |
| TCGA-64-1678-01A-01R-0946-07 | Alive | 1189 | 0.5983 |
| TCGA-64-1679-01A-21R-2066-07 | Alive | 2488 | 0.6318 |
| TCGA-64-1680-01A-02R-0946-07 | Alive | 1126 | 0.3481 |
| TCGA-64-1681-01A-11R-2066-07 | Dead | 1167 | 0.3596 |
| TCGA-64-5774-01A-01R-1628-07 | Alive | 2676 | 1.6846 |
| TCGA-64-5775-01A-01R-1628-07 | Dead | 62 | 1.2071 |
| TCGA-64-5778-01A-01R-1628-07 | Alive | 1305 | 1.0811 |
| TCGA-64-5779-01A-01R-1628-07 | Alive | 864 | 0.3052 |
| TCGA-64-5781-01A-01R-1628-07 | Alive | 1559 | 0.6145 |
| TCGA-64-5815-01A-01R-1628-07 | Alive | 866 | 1.7135 |
| TCGA-67-3770-01A-01R-0946-07 | Alive | 610 | 0.3182 |
| TCGA-67-3771-01A-01R-0946-07 | Alive | 610 | 0.1658 |
| TCGA-67-3772-01A-01R-0946-07 | Alive | 573 | 0.4665 |
| TCGA-67-3773-01A-01R-0946-07 | Alive | 427 | 0.1693 |
| TCGA-67-3774-01A-01R-0946-07 | Alive | 385 | 0.2429 |
| TCGA-67-4679-01B-01R-1755-07 | Alive | 448 | 0.0431 |
| TCGA-67-6215-01A-11R-1755-07 | Alive | 174 | 0.3537 |
| TCGA-67-6216-01A-11R-1755-07 | Alive | 141 | 1.4286 |
| TCGA-67-6217-01A-11R-1755-07 | Alive | 422 | 0.2931 |
| TCGA-69-7760-01A-11R-2170-07 | Alive | 202 | 1.3528 |
| TCGA-69-7761-01A-11R-2170-07 | Alive | 186 | 0.9142 |
| TCGA-69-7763-01A-11R-2170-07 | Alive | 690 | 0.4199 |
| TCGA-69-7764-01A-11R-2170-07 | Alive | 414 | 0.1927 |
| TCGA-69-7765-01A-11R-2170-07 | Alive | 165 | 0.9855 |
| TCGA-69-7973-01A-11R-2187-07 | Alive | 230 | 0.7071 |
| TCGA-69-7974-01A-11R-2187-07 | Alive | 184 | 1.0431 |
| TCGA-69-7978-01A-11R-2187-07 | Alive | 134 | 1.1982 |
| TCGA-69-7979-01A-11R-2187-07 | Alive | 408 | 0.3299 |
| TCGA-69-7980-01A-11R-2187-07 | Alive | 411 | 0.4908 |
| TCGA-69-8253-01A-11R-2287-07 | Alive | 426 | 0.3066 |
| TCGA-69-8254-01A-11R-2287-07 | Alive | 409 | 1.4065 |
| TCGA-69-8255-01A-11R-2287-07 | Alive | 129 | 0.2000 |
| TCGA-69-8453-01A-12R-2326-07 | Alive | 813 | 0.6040 |
| TCGA-69-A59K-01A-11R-A262-07 | Alive | 591 | 1.9608 |
| TCGA-71-6725-01A-11R-1858-07 | Alive | 256 | 0.2983 |
| TCGA-71-8520-01A-11R-2403-07 | Dead | 210 | 0.8712 |
| TCGA-73-4658-01A-01R-1755-07 | Dead | 1600 | 0.4629 |
| TCGA-73-4659-01A-01R-1206-07 | Dead | 711 | 0.1923 |
| TCGA-73-4662-01A-01R-1206-07 | Alive | 2515 | 0.5476 |
| TCGA-73-4666-01A-01R-1206-07 | Alive | 800 | 1.7151 |
| TCGA-73-4668-01A-01R-1206-07 | Alive | 467 | 1.3491 |
| TCGA-73-4670-01A-01R-1206-07 | Alive | 131 | 1.5809 |
| TCGA-73-4675-01A-01R-1206-07 | Dead | 922 | 0.1202 |
| TCGA-73-4676-01A-01R-1755-07 | Dead | 281 | 1.1691 |
| TCGA-73-4677-01A-01R-1206-07 | Dead | 38 | 0.1209 |
| TCGA-73-7498-01A-12R-2187-07 | Alive | 1189 | 0.7256 |
| TCGA-73-7499-01A-11R-2187-07 | Dead | 1531 | 0.6906 |
| TCGA-73-A9RS-01A-11R-A41B-07 | Dead | 340 | 0.1073 |
| TCGA-75-5122-01A-01R-1755-07 | Dead |  | 0.1120 |
| TCGA-75-5125-01A-01R-1755-07 | Dead | 2027 | 2.1933 |
| TCGA-75-5126-01A-01R-1755-07 | Alive |  | 1.1351 |
| TCGA-75-5146-01A-01R-1628-07 | Alive | 2368 | 0.1086 |
| TCGA-75-5147-01A-01R-1628-07 | Alive | 1333 | 1.0098 |
| TCGA-75-6203-01A-11R-1755-07 | Alive |  | 0.0690 |
| TCGA-75-6205-01A-11R-1755-07 | Dead |  | 0.2927 |
| TCGA-75-6206-01A-11R-1755-07 | Alive | 2590 | 0.3667 |
| TCGA-75-6207-01A-11R-1755-07 | Dead |  | 2.3772 |
| TCGA-75-6211-01A-11R-1755-07 | Dead |  | 1.4214 |
| TCGA-75-6212-01A-11R-1755-07 | Dead | 1516 | 0.2783 |
| TCGA-75-6214-01A-41R-1949-07 | Dead | 1115 | 2.6449 |
| TCGA-75-7025-01A-12R-1949-07 | Alive | 3305 | 0.6579 |
| TCGA-75-7027-01A-11R-1949-07 | Alive | 3059 | 1.5361 |
| TCGA-75-7030-01A-11R-1949-07 | Alive |  | 1.0387 |
| TCGA-75-7031-01A-11R-1949-07 | Alive |  | 0.4515 |
| TCGA-78-7143-01A-11R-2039-07 | Dead | 4961 | 0.7933 |
| TCGA-78-7145-01A-11R-2039-07 | Dead | 826 | 1.0282 |
| TCGA-78-7146-01A-11R-2039-07 | Dead | 173 | 2.5083 |
| TCGA-78-7147-01A-11R-2039-07 | Dead | 586 | 0.4361 |
| TCGA-78-7148-01A-11R-2039-07 | Dead | 626 | 0.7057 |
| TCGA-78-7149-01A-11R-2039-07 | Alive | 3940 | 0.2338 |
| TCGA-78-7150-01A-21R-2039-07 | Dead | 666 | 0.6792 |
| TCGA-78-7152-01A-11R-2039-07 | Dead | 1215 | 0.3474 |
| TCGA-78-7153-01A-11R-2039-07 | Alive | 3635 | 0.1601 |
| TCGA-78-7154-01A-11R-2039-07 | Dead | 593 | 0.9816 |
| TCGA-78-7155-01A-11R-2039-07 | Dead | 1171 | 0.0000 |
| TCGA-78-7156-01A-11R-2039-07 | Dead | 976 | 0.0727 |
| TCGA-78-7158-01A-11R-2039-07 | Dead | 179 | 0.7120 |
| TCGA-78-7159-01A-11R-2039-07 | Alive | 1974 | 0.4193 |
| TCGA-78-7160-01A-11R-2039-07 | Dead | 697 | 0.9460 |
| TCGA-78-7161-01A-11R-2039-07 | Dead | 291 | 1.3240 |
| TCGA-78-7162-01A-21R-2066-07 | Dead | 3169 | 0.0148 |
| TCGA-78-7163-01A-12R-2066-07 | Alive | 7248 | 0.6835 |
| TCGA-78-7166-01A-12R-2066-07 | Dead | 258 | 0.8087 |
| TCGA-78-7167-01A-11R-2066-07 | Dead | 2681 | 0.5242 |
| TCGA-78-7220-01A-11R-2039-07 | Dead | 807 | 0.4675 |
| TCGA-78-7535-01A-11R-2066-07 | Dead | 949 | 0.3827 |
| TCGA-78-7536-01A-11R-2066-07 | Dead | 244 | 0.2438 |
| TCGA-78-7537-01A-11R-2066-07 | Dead | 1622 | 0.0683 |
| TCGA-78-7539-01A-11R-2066-07 | Alive | 791 | 0.0801 |
| TCGA-78-7540-01A-11R-2066-07 | Dead | 1197 | 0.6986 |
| TCGA-78-7542-01A-21R-2066-07 | Dead | 321 | 2.0721 |
| TCGA-78-7633-01A-11R-2066-07 | Dead | 1528 | 0.1304 |
| TCGA-78-8640-01A-11R-2403-07 | Alive | 7062 | 0.0216 |
| TCGA-78-8648-01A-11R-2403-07 | Dead | 1209 | 0.1618 |
| TCGA-78-8655-01A-11R-2403-07 | Alive | 2360 | 0.5422 |
| TCGA-78-8660-01A-11R-2403-07 | Dead | 321 | 1.5691 |
| TCGA-78-8662-01A-11R-2403-07 | Dead | 3361 | 0.0000 |
| TCGA-80-5607-01A-31R-1949-07 | Alive |  | 0.4990 |
| TCGA-80-5608-01A-31R-1949-07 | Alive | 2832 | 0.9418 |
| TCGA-80-5611-01A-01R-1628-07 | Alive | 2595 | 0.2680 |
| TCGA-83-5908-01A-21R-2287-07 | Alive | 824 | 2.0528 |
| TCGA-86-6562-01A-11R-1755-07 | Dead | 376 | 1.5386 |
| TCGA-86-6851-01A-11R-1949-07 | Alive | 179 | 1.1472 |
| TCGA-86-7701-01A-11R-2170-07 | Alive | 947 | 1.8026 |
| TCGA-86-7711-01A-11R-2066-07 | Dead | 1046 | 0.0108 |
| TCGA-86-7713-01A-11R-2066-07 | Alive | 1157 | 0.6240 |
| TCGA-86-7714-01A-12R-2170-07 | Dead | 625 | 0.9716 |
| TCGA-86-7953-01A-11R-2187-07 | Alive | 997 | 0.4514 |
| TCGA-86-7954-01A-11R-2187-07 | Alive | 605 | 0.5396 |
| TCGA-86-7955-01A-11R-2187-07 | Alive | 1072 | 1.3359 |
| TCGA-86-8054-01A-11R-2241-07 | Alive | 1148 | 0.3783 |
| TCGA-86-8055-01A-11R-2241-07 | Dead | 124 | 0.5601 |
| TCGA-86-8056-01A-11R-2241-07 | Alive | 139 | 0.1601 |
| TCGA-86-8073-01A-11R-2241-07 | Alive | 740 | 0.5052 |
| TCGA-86-8074-01A-11R-2241-07 | Alive | 24 | 1.6524 |
| TCGA-86-8075-01A-11R-2241-07 | Dead | 694 | 0.4405 |
| TCGA-86-8076-01A-31R-2241-07 | Alive | 993 | 0.4026 |
| TCGA-86-8278-01A-11R-2287-07 | Alive | 944 | 0.6961 |
| TCGA-86-8279-01A-11R-2287-07 | Alive | 949 | 0.2126 |
| TCGA-86-8280-01A-11R-2287-07 | Alive | 701 | 0.4919 |
| TCGA-86-8281-01A-11R-2287-07 | Alive | 0 | 0.3833 |
| TCGA-86-8358-01A-11R-2326-07 | Alive | 653 | 0.7205 |
| TCGA-86-8359-01A-11R-2326-07 | Dead | 444 | 0.4151 |
| TCGA-86-8585-01A-11R-2403-07 | Alive | 353 | 1.9288 |
| TCGA-86-8668-01A-11R-2403-07 | Alive | 423 | 0.1582 |
| TCGA-86-8669-01A-11R-2403-07 | Alive | 938 | 0.4919 |
| TCGA-86-8671-01A-11R-2403-07 | Alive | 839 | 0.3906 |
| TCGA-86-8672-01A-21R-2403-07 | Dead | 19 | 0.8525 |
| TCGA-86-8673-01A-11R-2403-07 | Alive | 862 | 0.7283 |
| TCGA-86-8674-01A-21R-2403-07 | Alive | 806 | 0.2925 |
| TCGA-86-A456-01A-11R-A24H-07 | Alive | 896 | 0.7817 |
| TCGA-86-A4D0-01A-11R-A24H-07 | Dead | 116 | 0.9413 |
| TCGA-86-A4JF-01A-11R-A24X-07 | Dead | 737 | 0.5071 |
| TCGA-86-A4P7-01A-11R-A24X-07 | Alive | 415 | 0.2801 |
| TCGA-86-A4P8-01A-11R-A24X-07 | Alive | 805 | 0.0578 |
| TCGA-91-6828-01A-11R-1858-07 | Alive | 323 | 0.6377 |
| TCGA-91-6829-01A-21R-1858-07 | Dead | 1258 | 0.5277 |
| TCGA-91-6830-01A-11R-1949-07 | Alive | 60 | 1.1686 |
| TCGA-91-6831-01A-11R-1858-07 | Alive | 310 | 0.2196 |
| TCGA-91-6835-01A-11R-1858-07 | Alive | 79 | 0.3985 |
| TCGA-91-6836-01A-21R-1858-07 | Alive | 417 | 3.3266 |
| TCGA-91-6840-01A-11R-1949-07 | Alive | 372 | 0.0686 |
| TCGA-91-6847-01A-11R-1949-07 | Alive | 842 | 1.9073 |
| TCGA-91-6848-01A-11R-1949-07 | Alive | 224 | 1.1630 |
| TCGA-91-6849-01A-11R-1949-07 | Alive | 35 | 0.1219 |
| TCGA-91-7771-01A-11R-2170-07 | Alive | 492 | 0.3366 |
| TCGA-91-8496-01A-11R-2403-07 | Alive | 505 | 0.0482 |
| TCGA-91-8497-01A-11R-2403-07 | Dead | 434 | 0.2455 |
| TCGA-91-8499-01A-11R-2403-07 | Alive | 36 | 0.1140 |
| TCGA-91-A4BC-01A-11R-A24H-07 | Alive | 44 | 0.3742 |
| TCGA-91-A4BD-01A-11R-A24H-07 | Alive | 603 | 0.3173 |
| TCGA-93-7347-01A-11R-2187-07 | Alive | 683 | 0.4806 |
| TCGA-93-7348-01A-21R-2039-07 | Alive | 531 | 0.4584 |
| TCGA-93-8067-01A-11R-2287-07 | Alive | 186 | 0.9325 |
| TCGA-93-A4JN-01A-11R-A24X-07 | Alive | 718 | 0.5245 |
| TCGA-93-A4JO-01A-21R-A24X-07 | Dead | 33 | 0.0946 |
| TCGA-93-A4JP-01A-11R-A24X-07 | Alive | 578 | 0.4852 |
| TCGA-93-A4JQ-01A-11R-A24X-07 | Alive | 526 | 0.7735 |
| TCGA-95-7039-01A-11R-1949-07 | Alive | 1272 | 1.1955 |
| TCGA-95-7043-01A-11R-1949-07 | Dead | 503 | 0.1753 |
| TCGA-95-7562-01A-11R-2241-07 | Dead | 87 | 0.9772 |
| TCGA-95-7567-01A-11R-2066-07 | Alive | 568 | 1.2372 |
| TCGA-95-7944-01A-11R-2187-07 | Alive | 377 | 0.1174 |
| TCGA-95-7947-01A-11R-2187-07 | Alive | 477 | 0.2662 |
| TCGA-95-7948-01A-11R-2187-07 | Alive | 476 | 0.6465 |
| TCGA-95-8039-01A-11R-2241-07 | Alive | 830 | 0.5934 |
| TCGA-95-8494-01A-11R-2326-07 | Alive | 84 | 3.0570 |
| TCGA-95-A4VK-01A-11R-A262-07 | Alive | 651 | 0.7511 |
| TCGA-95-A4VN-01A-11R-A262-07 | Alive | 553 | 4.0554 |
| TCGA-95-A4VP-01A-21R-A262-07 | Alive | 605 | 0.9322 |
| TCGA-97-7546-01A-11R-2039-07 | Alive | 1285 | 0.0823 |
| TCGA-97-7547-01A-11R-2039-07 | Alive | 1965 | 0.1524 |
| TCGA-97-7552-01A-11R-2039-07 | Alive | 1932 | 0.4755 |
| TCGA-97-7553-01A-21R-2039-07 | Alive | 1870 | 0.8986 |
| TCGA-97-7554-01A-11R-2039-07 | Alive | 775 | 1.3220 |
| TCGA-97-7937-01A-11R-2170-07 | Alive | 564 | 0.2580 |
| TCGA-97-7938-01A-11R-2170-07 | Dead | 18 | 0.1666 |
| TCGA-97-7941-01A-11R-2187-07 | Alive | 484 | 1.2207 |
| TCGA-97-8171-01A-11R-2287-07 | Alive | 568 | 0.2614 |
| TCGA-97-8172-01A-11R-2287-07 | Alive | 545 | 0.1326 |
| TCGA-97-8174-01A-11R-2287-07 | Dead | 164 | 0.1213 |
| TCGA-97-8175-01A-11R-2287-07 | Alive | 551 | 0.7486 |
| TCGA-97-8176-01A-11R-2403-07 | Dead | 468 | 1.1442 |
| TCGA-97-8177-01A-11R-2287-07 | Alive | 499 | 0.0934 |
| TCGA-97-8179-01A-11R-2287-07 | Alive | 435 | 2.4353 |
| TCGA-97-8547-01A-11R-2403-07 | Alive | 657 | 2.0661 |
| TCGA-97-8552-01A-11R-2403-07 | Alive | 626 | 0.2003 |
| TCGA-97-A4LX-01A-11R-A24X-07 | Alive | 614 | 1.9466 |
| TCGA-97-A4M0-01A-11R-A24X-07 | Alive | 652 | 1.1257 |
| TCGA-97-A4M1-01A-11R-A24X-07 | Alive | 601 | 0.0726 |
| TCGA-97-A4M2-01A-12R-A24X-07 | Alive | 624 | 0.2260 |
| TCGA-97-A4M3-01A-11R-A24X-07 | Alive | 540 | 0.6793 |
| TCGA-97-A4M5-01A-11R-A24X-07 | Alive | 634 | 0.5345 |
| TCGA-97-A4M6-01A-11R-A24X-07 | Alive | 568 | 0.4916 |
| TCGA-97-A4M7-01A-11R-A24X-07 | Alive | 629 | 0.3925 |
| TCGA-99-7458-01A-11R-2039-07 | Alive | 747 | 1.3678 |
| TCGA-99-8025-01A-11R-2241-07 | Alive | 1060 | 0.8510 |
| TCGA-99-8028-01A-11R-2241-07 | Alive | 1118 | 1.3002 |
| TCGA-99-8032-01A-11R-2241-07 | Alive | 44 | 1.0461 |
| TCGA-99-8033-01A-11R-2241-07 | Dead | 656 | 0.6809 |
| TCGA-99-AA5R-01A-11R-A39D-07 | Alive | 658 | 0.2448 |
| TCGA-J2-8192-01A-11R-2241-07 | Alive | 739 | 1.6668 |
| TCGA-J2-8194-01A-11R-2241-07 | Alive | 724 | 0.8240 |
| TCGA-J2-A4AD-01A-11R-A24H-07 | Dead | 550 | 1.0119 |
| TCGA-J2-A4AE-01A-21R-A24H-07 | Alive | 1079 | 0.2984 |
| TCGA-J2-A4AG-01A-11R-A24H-07 | Alive | 988 | 0.5441 |
| TCGA-L4-A4E5-01A-11R-A24X-07 | Alive | 578 | 2.7310 |
| TCGA-L4-A4E6-01A-11R-A24H-07 | Alive | 435 | 0.3778 |
| TCGA-L9-A443-01A-12R-A24H-07 | Dead | 193 | 1.3343 |
| TCGA-L9-A444-01A-21R-A24H-07 | Alive | 307 | 0.7461 |
| TCGA-L9-A50W-01A-12R-A39D-07 | Dead | 442 | 2.0470 |
| TCGA-L9-A5IP-01A-21R-A39D-07 | Dead | 58 | 1.8620 |
| TCGA-L9-A743-01A-43R-A39D-07 | Alive | 664 | 0.5481 |
| TCGA-L9-A7SV-01A-11R-A39D-07 | Alive | 565 | 1.7737 |
| TCGA-L9-A8F4-01A-11R-A39D-07 | Alive | 476 | 0.7509 |
| TCGA-MN-A4N1-01A-11R-A24X-07 | Alive | 827 | 1.9664 |
| TCGA-MN-A4N4-01A-12R-A24X-07 | Alive | 1175 | 0.6102 |
| TCGA-MN-A4N5-01A-11R-A24X-07 | Alive | 84 | 2.5073 |
| TCGA-MP-A4SV-01A-11R-A24X-07 | Dead | 2620 | 1.6854 |
| TCGA-MP-A4SW-01A-21R-A24X-07 | Dead | 1778 | 1.3840 |
| TCGA-MP-A4SY-01A-21R-A24X-07 | Dead | 1501 | 1.2986 |
| TCGA-MP-A4T4-01A-11R-A262-07 | Dead | 2617 | 1.7259 |
| TCGA-MP-A4T6-01A-32R-A262-07 | Dead | 1790 | 0.9395 |
| TCGA-MP-A4T7-01A-11R-A24X-07 | Dead | 167 | 0.9534 |
| TCGA-MP-A4T8-01A-11R-A24X-07 | Dead | 161 | 1.8425 |
| TCGA-MP-A4T9-01A-11R-A24X-07 | Dead | 1265 | 1.4120 |
| TCGA-MP-A4TA-01A-21R-A24X-07 | Dead | 950 | 1.1868 |
| TCGA-MP-A4TC-01A-11R-A24X-07 | Dead | 74 | 0.7292 |
| TCGA-MP-A4TD-01A-32R-A262-07 | Dead | 307 | 0.8597 |
| TCGA-MP-A4TE-01A-22R-A466-07 | Dead | 896 | 1.1582 |
| TCGA-MP-A4TF-01A-11R-A262-07 | Dead | 336 | 3.1019 |
| TCGA-MP-A4TH-01A-31R-A262-07 | Alive | 741 | 0.2405 |
| TCGA-MP-A4TI-01A-21R-A24X-07 | Dead | 429 | 2.4707 |
| TCGA-MP-A4TJ-01A-51R-A262-07 | Dead | 339 | 0.1521 |
| TCGA-MP-A4TK-01A-11R-A24X-07 | Dead | 582 | 1.6172 |
| TCGA-MP-A5C7-01A-11R-A262-07 | Alive | 2248 | 0.1652 |
| TCGA-NJ-A4YF-01A-12R-A262-07 | Alive | 2161 | 1.0475 |
| TCGA-NJ-A4YG-01A-22R-A262-07 | Alive | 2261 | 1.2119 |
| TCGA-NJ-A4YI-01A-11R-A262-07 | Dead | 4 | 0.7127 |
| TCGA-NJ-A4YP-01A-11R-A262-07 | Alive | 50 | 1.0433 |
| TCGA-NJ-A4YQ-01A-11R-A262-07 | Alive | 1432 | 0.1379 |
| TCGA-NJ-A55A-01A-11R-A262-07 | Alive | 15 | 0.2937 |
| TCGA-NJ-A55O-01A-11R-A262-07 | Alive | 13 | 0.8934 |
| TCGA-NJ-A55R-01A-11R-A262-07 | Alive | 603 | 2.0224 |
| TCGA-NJ-A7XG-01A-12R-A39D-07 | Alive | 617 | 0.8097 |
| TCGA-O1-A52J-01A-11R-A262-07 | Dead | 1798 | 0.2494 |
| TCGA-S2-AA1A-01A-12R-A39D-07 | Alive | 513 | 0.1965 |
